# Fasting for stem cell rejuvenation

**DOI:** 10.18632/aging.102912

**Published:** 2020-03-06

**Authors:** Cristina González-Estévez, Ignacio Flores

**Affiliations:** 1 Leibniz Institute on Aging-Fritz Lipmann Institute (FLI), Jena 07745, Germany; 2 Centro Nacional de Investigaciones Cardiovasculares Carlos III (CNIC), Madrid 28029, Spain; 3 Centro de Biologia Molecular Severo Ochoa, CSIC-UAM, Cantoblanco, Madrid, Spain

**Keywords:** planarian, mTOR, *Schmidtea mediterranea*, starvation, stem cell, telomere

Throughout the centuries, humankind has relentlessly searched for ways to live longer and healthier lives. From the "fountain of youth" quest to novel senolytics, from alchemical recipes to modern diets, different approaches have been pursued to fulfil the human desire of prolonging life while maintaining good shape. Among all anti-aging interventions, calorie restricted diets and periods of fasting stand out as the most compelling and robust methods to prolong life and health span and to reduce the risk of diabetes, neurodegeneration, autoimmune disorders, spontaneous tumours and cardiovascular disease [1]. Furthermore, dietary interventions are also emerging as important enhancers of adult stem cell function [2]. However, little is known on how prolonged fasting alters the function and properties of adult stem cells. Since fasting outcomes are conserved across taxa [2], studying fasting in species that possess many stem cells and can cope with long periods of food deprivation can be exceedingly informative.

Planarians -better known for their impressive regenerative capacities- can be deprived of food for more than 3 months without showing an impairment in either physiology or activity levels. They handle prolonged periods of starvation or fasting by shrinking in size. Around 25% of the cells in their parenchyma are adult stem cells, which are kept in a constant ration respect their body size. Interestingly, refeeding allows fasted planarians to grow back to their original size [3]. Their stem cells do not show any signs of senescence and hence they are considered immortal. How fasting influences planarian stem cell properties is unknown.

**Figure 1:**
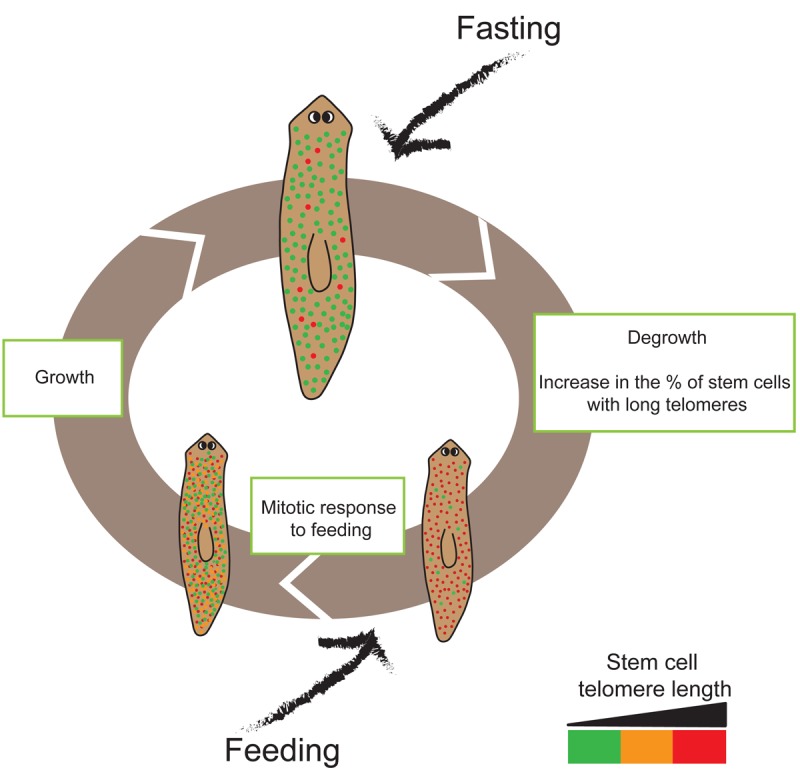
**Schematic representation of the life cycle of *Schmidtea mediterranea* asexual strain.** Cycles of feeding and fasting are common during planarian life. During fasting the percentage of stem cells with long telomeres increases. Feeding induces a rapid proliferative response. During growing due to feeding, the stem cell pool decreases its telomere length. Red cells indicate stem cells with the longest telomeres while the orange show medium length and the green ones the shortest telomeres. Planarians are not to scale.

We have recently reported the effect of fasting on planarian stem cells regarding telomere length [4]. Telomeres protect chromosomes from DNA degradation and misguided repair mechanisms. Proper telomere functioning requires a minimum length that is maintain by telomerase. However, telomerase activity levels in adult tissues are not sufficient to prevent progressive telomere shortening with age [5]. Therefore, telomere length is considered a cellular marker of aging. By measuring telomere length *in situ* on whole planarians we found that fasted planarians present a higher percentage of stem cells with the longest telomeres, indicating that fasting rejuvenates the stem cell pool [4]. Having a population of stem cells with very long telomeres allows planarians to quickly respond to any injury even while fasting. It also allows them to mount a long-term proliferation response as soon as nutrients become available again. Therefore, natural cycles of fasting and feeding promote the maintenance of a healthy and always cycling stem cell population thus making planarians immortal (Figure 1).

Our data shows that the enrichment of stem cells with long telomeres during fasting occurs through the inhibition of mTOR signalling [4], a pathway known to enhance stem cell function during dietary restriction [2]. The easy explanation to understand how mTOR down-regulation elongates telomeres is through a reduction in mitosis. It is indeed known that mTOR signalling regulates the mitotic response to amputation and blastema growth [6]. However, while fasting increases telomere length, the number of mitosis and stem cells remains constant [7]. Other factors than cell division may modulate telomere length, for instance exonucleases or oxygen levels [5]. It is also feasible that stem cells with the shortest telomeres are considered “less-fit” or “loser”, being selected to either die or differentiate and contributing in this way to a general increase in telomere length in the remaining stem cell pool. Interestingly, mTOR signalling has been linked to “cell competition” and an mTOR-controlled process, autophagy, has been shown to be required by “loser” cells to die [8]. The question still remains on whether fasting affects other molecular/cellular processes in planarian stem cells. Ongoing research will clarify this point.

Both activation of telomerase and long telomere length are known to positively correlate with stem cell pluripotency. Interestingly we find that the stem cell population is highly heterogeneous for telomere length, correlating with their known heterogeneity with regards to potency and lineage commitment [4]. We also find that fasting not only increases the percentage of stem cells with long telomeres but also increases the maximum telomere length in planarian stem cells [4]. Altogether leads to the attractive hypothesis that fasting, by modulating mTOR signalling, may increase pluripotency in planarians. Our work opens up many interesting endeavours which we predict will help in the understanding of regeneration and stem cell ageing.
